# 

**Published:** 1970-01

**Authors:** 


NATIONAL LIBRARY OF MEDICINE

RECD. FEB 12 1970

INDEX FEB 20 1970

INPUT

JTC: ASG
INDEXER: 39
VOL-ISS: 85-313
DATE: JAN 70
PRIORITY: 3

### BRISTOL MEDICO-CHIRURGICAL JOURNAL

NATIONAL LIBRARY OF MEDICINE
INDEX COPY
RECD. FEB 16 1970
INDEX
INPUT MAR 5 1970

JANUARY 1970

VOL. 85 (I) No. 313

